# Ultrafast extreme ultraviolet photoemission without space charge

**DOI:** 10.1063/1.5045578

**Published:** 2018-09-06

**Authors:** Christopher Corder, Peng Zhao, Jin Bakalis, Xinlong Li, Matthew D. Kershis, Amanda R. Muraca, Michael G. White, Thomas K. Allison

**Affiliations:** 1 Stony Brook University, Stony Brook, New York 11794-3400, USA; 2 Brookhaven National Laboratory, Upton, New York 11973, USA

## Abstract

Time- and Angle-resolved photoelectron spectroscopy from surfaces can be used to record the dynamics of electrons and holes in condensed matter on ultrafast time scales. However, ultrafast photoemission experiments using extreme-ultraviolet (XUV) light have previously been limited by either space-charge effects, low photon flux, or limited tuning range. In this article, we describe XUV photoelectron spectroscopy experiments with up to 5 nA of average sample current using a tunable cavity-enhanced high-harmonic source operating at 88 MHz repetition rate. The source delivers >10^11^ photons/s in isolated harmonics to the sample over a broad photon energy range from 18 to 37 eV with a spot size of 58 × 100 *μ*m^2^. From photoelectron spectroscopy data, we place conservative upper limits on the XUV pulse duration and photon energy bandwidth of 93 fs and 65 meV, respectively. The high photocurrent, lack of strong space charge distortions of the photoelectron spectra, and excellent isolation of individual harmonic orders allow us to observe laser-induced modifications of the photoelectron spectra at the 10^−4^ level, enabling time-resolved XUV photoemission experiments in a qualitatively new regime.

## I. INTRODUCTION

Angle-resolved photoelectron spectroscopy (ARPES) using synchrotron radiation has become an essential tool for condensed matter physics and surface science. The high spectral brightness of synchrotron radiation allows photoelectron spectra to be recorded with photocurrents in the nano-Ampere range. These large photocurrents, parsed by sophisticated electron energy analyzers,^1–3^ enable detailed studies of the electronic structure of solids and surfaces in energy, momentum, and spin. Tuning the photon energy throughout the extreme ultraviolet (XUV, 10–100 eV) allows experiments to map the energy dispersion relation for momentum perpendicular to the surface (*k_z_*), interpret the contributions of final state effects to the measured energy distribution curves (EDC), and choose between increased surface or bulk sensitivity.^4^

Shortly after the development of high-power femtosecond lasers and discovery of high-order harmonic generation (HHG), in which a broad range of laser-harmonics are coherently emitted from a field-ionized medium,^5^ HHG was applied to surface photoemission experiments.^6–8^ Indeed, the range of photon energies typically emitted from HHG driven by Ti:Sapphire lasers in noble gasses is nicely coincident with the range of photon energies used by ARPES beamlines at synchrotrons. In addition to the dramatically reduced cost and footprint compared to a synchrotron source, the HHG pulses had the advantage that they could be orders of magnitude shorter than the ∼100 ps pulse durations of synchrotrons, enabling ultrafast time-domain studies. However, it also became immediately apparent that photoemission experiments using HHG would be drastically limited compared to what is possible at synchrotrons.^8,9^

The principle limitation comes from the so-called “vacuum space-charge” effect.^10^ Consider a synchrotron experiment illuminating the sample with ∼10^12^ photons/s causing ∼10^11^ electrons/s (16 nA) to be emitted from the surface. The synchrotron photon pulses arrive at ∼100–1000 MHz repetition rate, so each burst of electrons emitted concurrently from a single pulse contains only ∼1000–100 electrons. In contrast, due to the high peak powers required to drive the HHG process efficiently, HHG is typically restricted to <100 kHz repetition rates. In order to maintain the same photocurrent, the electrons must then be concentrated by more than 1000 times, to more than 10^6^ electrons/pulse. The charging of space at these electron densities distorts the photoelectron spectrum on the eV energy scale, whereas synchrotron beamlines now routinely record photoelectron spectra with meV resolution.^11^ Practitioners of time-resolved photoelectron spectroscopy using HHG are then forced to compromise on the applied photon flux, focused spot size, resolution, fidelity of the signal, or some combination thereof.^9,12,13^

**FIG. 1.**
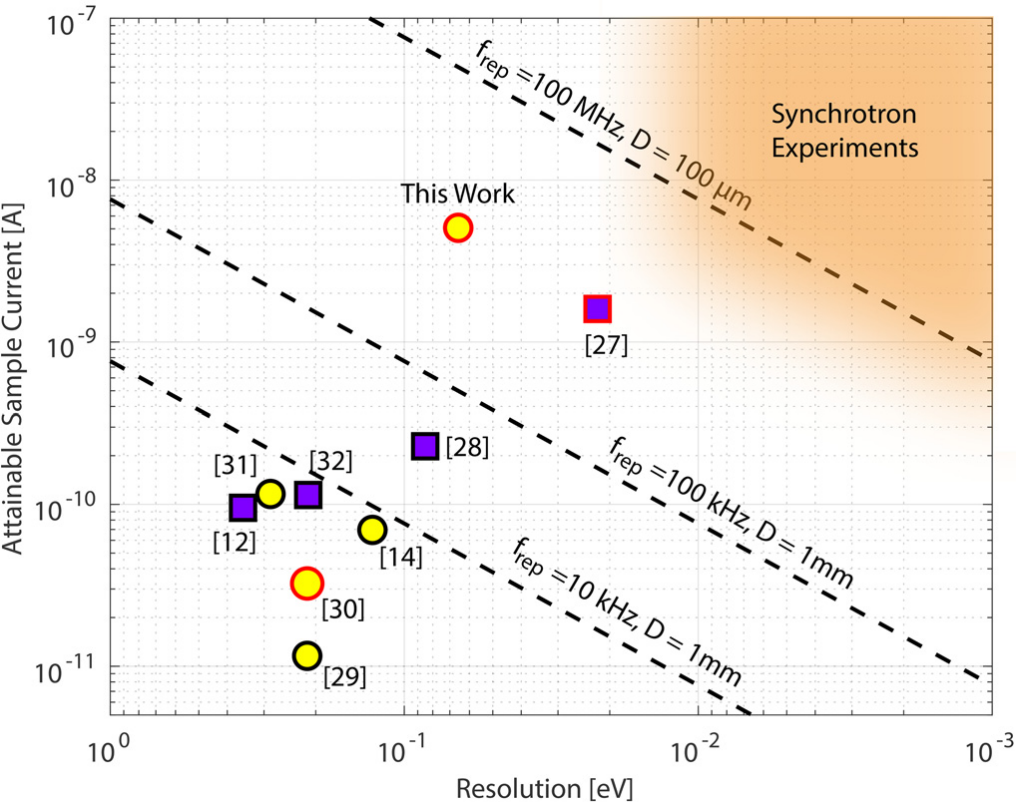
Constraints on sample current and energy resolution due to space charge effects. Dashed lines are created by evaluating Eq. (1) for different repetition rates by assuming 1 mm spot size and symbols represent published results applying HHG to surface photoemission. Yellow circles represent results from tunable HHG systems and purple squares represent setups where the photon energy is not tunable *in-situ*. Symbols with black edges represent space-charge limited spectrometers, and symbols with red edges represent systems that are not yet space-charge limited. For space-charge-limited systems, the (*x*, *y*) positions represent the case where space charge broadening and the photon bandwidth add equally in quadrature. See Appendix A for detailed explanation on how symbol placement was calculated based on published results.^12,14,27–32^

With the constraint of space charge setting the fundamental limits on the performance of photoemission experiments, this phenomenon has been extensively studied over a wide range of electron kinetic energies and pulse durations, both experimentally and theoretically.^9,10,12,14–18^ For sub-ps pulses and electron kinetic energies in the ∼5–100 eV range produced from conductive samples, both shifts and broadening of the photoelectron spectra features are observed to scale with linear electron density ρ≡N/D, where *N* is the number of electrons emitted from the sample per pulse and *D* is the spot size of the light on the sample. Expressed in terms of the average sample current (*I*_sample_) and repetition rate (*f*_rep_), this gives

$$\Delta E_{s,b}=m_{s,b}\frac{I_{\text{sample}}}{ef_{\text{rep}}D},$$ (1)

where $\Delta E_{s,b}$ is the energy shift (s) or energy broadening (b) of the photoelectron spectrum, *e* is the charge of the electron, and *m*_s,b_ are empirical scaling factors. Working in the extreme-ultraviolet (XUV), space charge effects do not depend strongly on the sample studied or the photon energy since the photoelectric yield is dominated by secondary electrons and the full Brillouin zone of inner valence bands. It is common practice to use the results obtained for simple metals in general considerations of the problem, and reports of the slope parameters *m*_s,b_ in the literature have varied by a factor of 2.^9,10,12,14,18^ Recently, using Cu (001), Plötzing *et al.*^12^ have studied these effects for multiple spot sizes and determined *m_b_* = 2.1 × 10^−6 ^eV mm and *m_s_* = 3.2 × 10^−6 ^eV mm with an estimated systematic uncertainty of less than 20%. Figure 1 illustrates the constraints on attainable sample current for a given resolution according to Eq. (1) using *m_b_* from Ref. 12. The dashed lines indicate the space charge limits for sub-ps laser-based systems of different repetition rates assuming a 1 mm spot size—large by ARPES standards. Even at the high repetition rate of 100 kHz and the coarse resolution of 100 meV, space charge constraints still limit the sample current to 760 pA.

For pump/probe experiments, these data-rate limitations are particularly stark for several reasons. First is that pump excitation adds additional dimensions to the data set. At a minimum, data should be recorded at several pump-probe delays and pump fluences, and there are also the parameters of pump wavelength and polarization. Second is that the signal of interest is inherently small, since only a fraction of the sample's electrons are excited by the pump. Combined, these two factors result in the need for orders of magnitude more data than ground state studies done at synchrotrons, but space charge limits the data rate to be orders of magnitude lower. Experiments have then been almost exclusively restricted to strongly excited samples using absorbed fluences on the order of ∼1 mJ/cm^2^,^19–21^ such that laser excitation produces changes to the EDC visible on a linear scale. In addition to probing different physics than can be accessed in the low-fluence regime,^22^ at high fluences ultrashort pump pulses also produce many electrons through multiphoton processes which add to the space-charge problem,^23–25^ and pump-induced space charge can also have a non-trivial dependence on the pump-probe delay, making space charge effects difficult to separate from the dynamics of interest.^26^

The inverse dependence of Eq. (1) on *f*_rep_ has motivated a great deal of work on high-power lasers and HHG source development. Although the highest HHG repetition rates have been reported using high-power frequency combs resonantly enhanced in optical cavities,^33,34^ their reliability and suitability for time-resolved photoemission have faced skepticism from several authors.^8,30,35^ Instead, there has been great investment in other approaches including HHG from high-power Ti:Sapphire and parametric amplifiers,^35,36^ HHG from high power fiber lasers,^37,38^ HHG generated within^39^ and at the output^40^ of thin-disk lasers, HHG from solids,^41,42^ and HHG in the near-fields of nanostructures.^43^ Despite these intense efforts, HHG-based photoemission comparable to that done with tunable synchrotron radiation has not been realized using any platform.

In this article, we demonstrate the application of a tunable cavity-enhanced HHG (CE-HHG) source, specially designed for this purpose, to the difficult problem of XUV photoemission. By performing experiments with high flux at 88 MHz repetition rate, nanoamperes of sample current can be generated from a sub-100 *μ*m laser spot with space charge effects estimated to be less than 10 meV, comparable to synchrotron-based ARPES experiments.^17,32^ We observe laser-induced modifications of the EDC at the 10^−4^ level in only minutes of integration time, demonstrating the feasibility of time-resolved ARPES measurements from perturbatively excited samples. In Sec. II, we describe critical and unique details of the light source along with its performance. In Sec. III, we demonstrate both static and time-resolved photoelectron spectroscopy measurements free of strong space-charge effects using nA sample currents. In Sec. IV, we compare this work to previous efforts and discuss how the system can be further improved.

## II. LIGHT SOURCE AND BEAMLINE

**FIG. 2.**
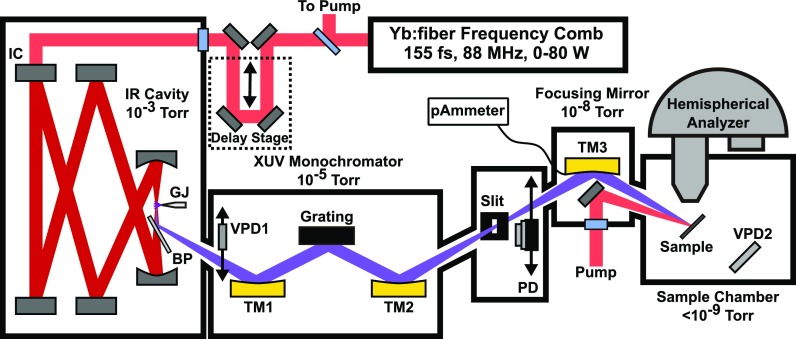
CE-HHG source and beamline. High-order harmonics of a resonantly enhanced Yb:fiber frequency comb are generated at the focus of a 6 mirror enhancement cavity and coupled into an XUV beamline. A pulse-preserving monochromator selects one harmonic which is focused on a sample under UHV conditions. BP = Brewster plate, VPD = vacuum photodiode, TM = toroidal mirror, PD = XUV photodiode, GJ = gas jet, IC = input coupler.

The experimental setup is shown in Fig. 2. A home-built 80 W, 155 fs frequency comb laser with a repetition rate of 88 MHz and a center wavelength of 1.035 *μ*m (*hν* = 1.2 eV) is passively amplified in a 6 mirror enhancement cavity with a 1% transmission input coupler and a finesse of F>560. We have described the laser in detail previously.^44^ The laser is locked to the cavity using a two-point Pound-Drever-Hall lock as described in Refs. 45 and 46. Harmonics are generated at a 24 *μ*m FWHM intracavity focus and reflected from a sapphire wafer placed at Brewster's angle for the resonant 1.035 *μ*m light. Noble gasses are injected to the focus using a fused silica capillary with a 100 *μ*m inside diameter. We optimize the nozzle position by moving it to maximize the photocurrent observed on a stainless steel vacuum photodiode (VPD1).^47^ Typical photocurrents from VPD1 are in the range of 100 to 300 nA. When generating harmonics, we also dose each intracavity optic with a mix of ozone and O_2_ from a commercial ozone generator to prevent hydrocarbon contamination, allowing continuous operation.

Typical intracavity powers for generating harmonics range from 5 to 11 kW, depending on the generating gas and desired harmonic spectrum, corresponding to intracavity peak intensities in the range of 0.6 to 1.3 × 10^14^ W/cm^2^. Intracavity nonlinear effects are observed from both the HHG gas and self-phase modulation in the Brewster plate, dropping the cavity's power enhancement and necessitating careful tuning of servo loop offsets.^46,48,49^ For example, the power enhancement drops from 270 at low power to 200 at 7.5 kW of intracavity power when generating harmonics in krypton.

The outcoupled harmonics are collimated by a 350 mm focal length toroidal mirror at 3° grazing angle (TM1) that forms the first part of a single off-plane grating pulse-preserving monochromator similar to the design of Frassetto *et al.*^50^ The harmonics strike a motorized grating at a 4° grazing angle and are refocused by a second *f *=* *350 mm toroidal mirror (TM2) at an adjustable slit. For all data presented here, the monochromator grating has 150 grooves/mm and is blazed for optimum diffraction efficiency for *λ* = 35 nm. With this grating, the monochromator selects an individual harmonic with tolerable pulse broadening but does not narrow the transmitted harmonic bandwidth. The exit slit plane of the monochromator is 1:1 imaged to the sample using another 350 mm focal length toroid at 3° grazing angle (TM3). Mirror TM3 is electrically floated such that the photocurrent of electrons ejected from the mirror surface can be used as a passive XUV intensity monitor. All beamline optics are gold coated and the XUV light is polarized perpendicular to the plane of incidence (s-polarization).

**FIG. 3.**
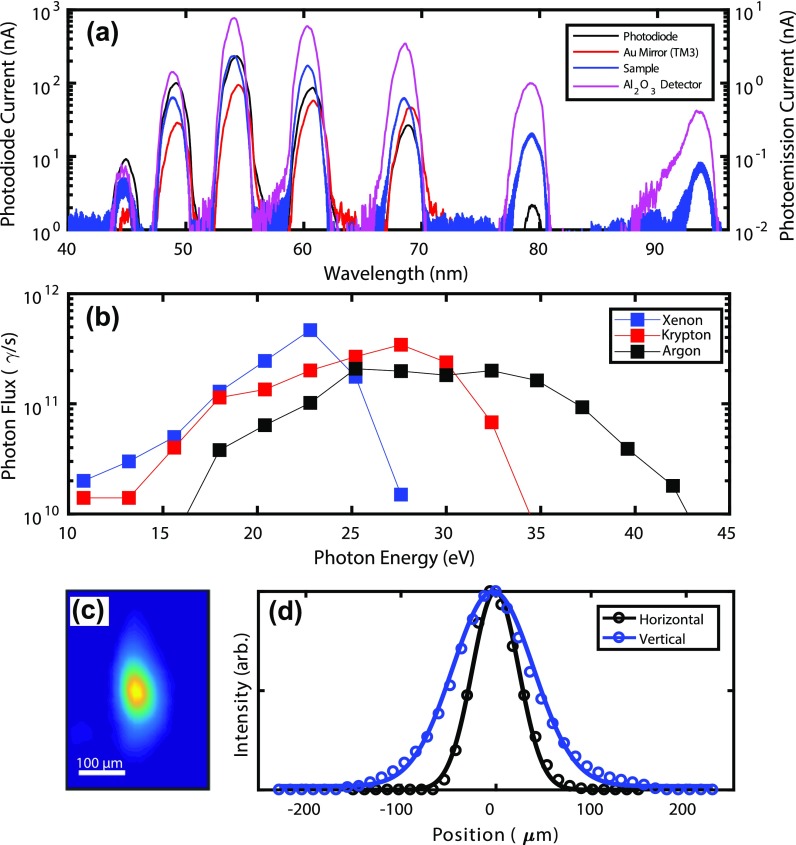
**(**a) An HHG spectrum from xenon gas measured by rotating the monochromator grating while using the four detectors after the exit slit. The photodiode current (black) uses the left y-axis, whereas the photoemission current from three downstream surfaces uses the right y-axis. Note that the harmonic linewidths are not resolved with the pulse-preserving monochromator design. (b) The photon flux delivered to the sample for each harmonic generated with the three gases. The flux has been calibrated using literature values for quantum efficiencies and no corrections for mirror losses have been made. (c) The 27th harmonic from Ar imaged with a Ce:YAG crystal at the sample position. (d) Lineouts through the centroid of (c) fit with Gaussian functions demonstrating 58 *μ*m × 100 *μ*m spot size (FWHM).

We detect the XUV flux exiting the monochromator and delivered to the sample using four separate detectors: an aluminum coated silicon photodiode (PD, Optodiode AXUV100Al), the photocurrent from TM3, the photocurrent from the sample, and the photocurrent from an Al_2_O_3_ vacuum photodiode^51^ (VPD2) placed at the end of the surface science chamber. Figure 3(a) shows a typical HHG spectrum from xenon gas measured using each of the four detectors as the monochromator grating is rotated. The observed harmonic linewidths in Fig. 3(b) are due to the intentionally small resolving power of the pulse-preserving monochromator, not the intrinsic harmonic linewidth.

The photon flux can be calculated using the measured photocurrent from all the detectors and literature values for the quantum efficiencies. All of these separate calculations agree within a factor of 2. Since contamination and surface oxidation only cause the quantum efficiency of XUV detectors to decrease, all calculated photon fluxes represent lower limits. In Fig. 3(b), the higher of the two lower limits from the PD or VPD2 are plotted as a function of photon energy and for three different generating gasses: argon, krypton, and xenon. As can be seen in Fig. 3(b), even using a single monochromator grating, by changing the generating gas, a flux of more than 10^11^ photons per second is delivered to the sample over a broad tuning range. At lower photon energies, the higher efficiency of generation in Kr and Xe compensates the reduced diffraction efficiency of the grating blazed for 35 eV. Higher fluxes can be obtained at lower photon energies using different gratings. For example, we have observed 7 × 10^11^ photons/s in the 21st harmonic from krypton (*hν* = 25.1 eV) using a 100 groove/mm grating blazed for 55 nm. These fluxes are within one order of magnitude of what is available from many state-of-the-art synchrotron beamlines dedicated to ARPES.^3,11,52^ Critically, since at 88 MHz, $7\times 10^{11}$ photons/s corresponds to only 8000 photons/pulse—also comparable to synchrotrons—all of this flux is usable for high-resolution photoemission experiments.

To measure the XUV spot at the sample, we image the fluorescence from a Ce:YAG scintillator plate placed at the sample position. Figure 3(c) shows the image and Fig. 3(d) shows Gaussian fits in the both the horizontal and vertical along lineouts intersecting the image centroid. The data indicate a clean elliptical beam with a FWHM of 58 *μ*m in the horizontal and 100 *μ*m in the vertical. Also, we measure that approximately 70% of the XUV light can be transmitted through a 100 *μ*m diameter pinhole oriented at 45° to the beam axis. This spot size is again similar to what is used at synchrotron beamlines.^11,52^ When comparing to previous HHG results, it is important to note that in our case this small spot size and high flux are actually usable for experiments due to the absence of space-charge effects at 88 MHz repetition rate. A small spot size enables studying spatially inhomogeneous samples (for example, produced by exfoliation^53^), requires less pump-pulse energy in pump/probe experiments, and is necessary for achieving high angular resolution in ARPES.

**FIG. 4.**
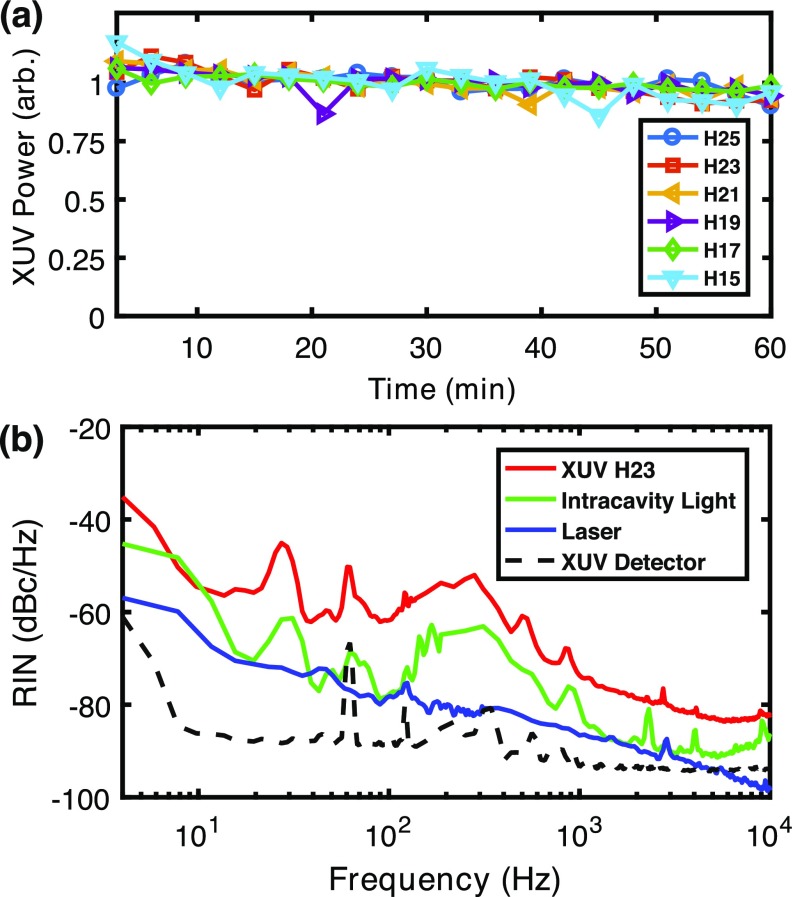
**(**a) Normalized XUV flux measured with PD after the monochromator for harmonics 15–25 from Kr over 1 h without human intervention. (b) Relative intensity noise (RIN) of the 23rd harmonic (red), intracavity laser light (green), and Yb:fiber laser (blue), along with the detector noise floor (black dashed).

We evaluated the amplitude noise and long term stability of the system using the PD detector. For the long term stability, we recorded a series of monochromator scans over a 1 h period without any human tuning of the laser alignment or servo loop. Figure 4(a) shows the relative power in the harmonics from Kr with a delivered flux greater than 10^11^ photons/s measured every 3 min. The RMS fluctuations averaged over all the harmonics over this period are 5%. Similar results are obtained for HHG in Ar or Xe as well. On longer time-scales, slow drifts in the laser alignment into the cavity and servo-loop offsets require occasional tuning to maintain the flux levels at those shown in Fig. 3(b). It is also important to note that since more than 100 pA of photocurrent is observed from TM3, drifts in the XUV flux can also be normalized using this *in-situ* monitor, as is commonly done at synchrotrons. At the time of writing, we have run the source on a near-daily basis without venting the vacuum system or performing any alignment of the in-vacuum optics for more than 2 months with stable and reproducible results, enabling the photoelectron spectroscopy experiments discussed in Sec. III.

For pump-probe experiments, it is often advantageous to use lock-in detection to extract small signals from large backgrounds. Figure 4(b) shows the amplitude noise (relative intensity noise, RIN) of the 23rd harmonic from Kr measured using the photodiode current amplified with a transimpedance amplifier and recorded with an FFT spectrum analyzer. For frequencies above 400 Hz, the RIN level is below −60 dBc/Hz, which can enable small differences in the photoelectron spectra to be recorded via lock-in detection. At this noise level, EDC or ARPES signals up to 10^6^ counts/s/bin can be photoelectron-shot-noise limited with proper correction for drift using the TM3 photocurrent.

## III. PHOTOEMISSION

Photoelectron spectroscopy measurements are performed under ultra-high vacuum conditions in a surface science endstation equipped with a hemispherical electron energy analyzer (VSW HA100). The analyzer is specified to have an angular acceptance of ±4° at the input and angle-integrated spectra are recorded from a channeltron detector at the exit as voltages scan the electron kinetic energy. The endstation is also equipped with a sputter gun, a LEED, a quadrupole mass spectrometer, an Al K*α* x-ray source, and a sample manipulator that can be cooled and heated between 100 and 1000 K. Also mounted on the sample manipulator are the Ce:YAG scintillator and pinhole mentioned in Sec. II. For all data presented here, the sample is oriented normal to the analyzer axis and 45° to the XUV beam. The electric field vector of the XUV light is in the plane of incidence (p-polarized) and the analyzer axis.

**FIG. 5.**
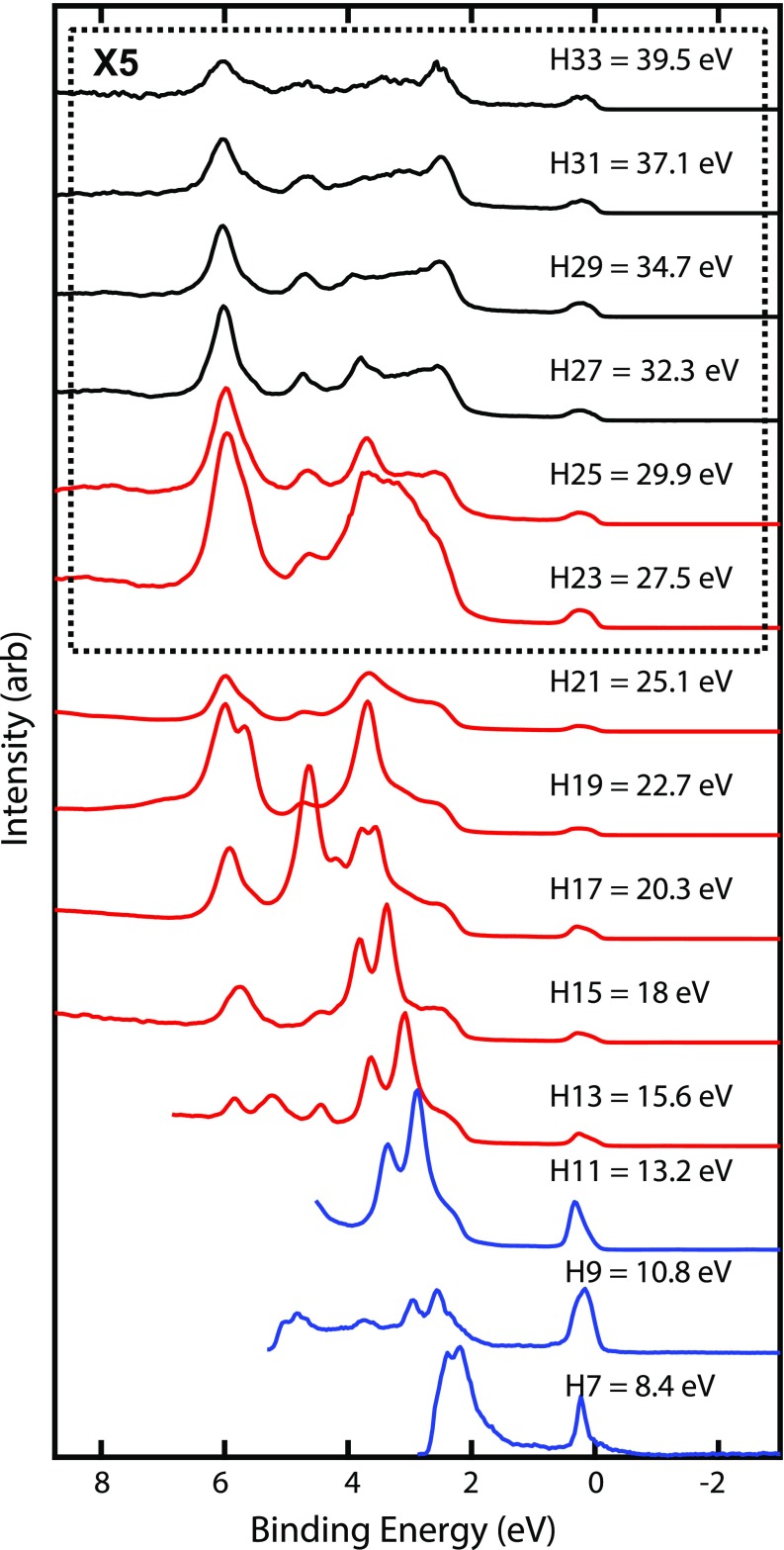
Static photoelectron spectra of an Au (111) surface taken with harmonics 7 through 33, vertically offset for clarity. The color indicates the gas used to generate the harmonic; Ar (black), Kr (red), and Xe (blue). Each EDC is normalized to the photocurrent measured at TM3, and spectra taken with photon energies above 25.1 eV have been enlarged by ×5.

Figure 5 shows photoelectron spectra from an Au (111) surface at 100 K temperature obtained using each harmonic between the 7th (*hν* = 8.4 eV) and 33rd (*hν* = 39.5 eV). Each spectrum was acquired with 34 meV steps individually measured with 1 s of integration for a total scan time of ∼6 min or less. At the d-band peaks, the electron count rates can exceed 1 MHz. These static spectra are in good agreement with those recorded by Kevan *et al.*^54,55^ using tunable synchrotron radiation. The clearly visible dispersion of the d-bands at binding energies between 3 and 7 eV and the large photon energy dependence of the relative amplitudes of the peaks highlight the importance of conducting photoemission experiments with a tunable source. The same final state effects also strongly influence time-resolved photoelectron spectra of excited states and tunability here should be considered no less important, as has been emphasized by previous authors.^56^

The resolution of the setup can be determined by analyzing the sharpness of the Fermi edge and is dominated by the energy analyzer. Fermi edge widths as low as 110 meV are measured depending on alignment. The best resolution we have been able to observe in any photoemission experiment using this analyzer is 89 meV using a He I lamp and a Kr gas target. From this data, we can place a conservative upper limit on the single harmonic photon energy bandwidth of $\sqrt{{\left(110\;\text{meV}\right)}^{2}-{\left(89\;\text{meV}\right)}^{2}}$ = 65 meV (details in supplementary material).

We have reason to believe that the single harmonic linewidth is lower than this. The single harmonic linewidth from HHG is determined by the duration of time over which harmonics are generated with comparable efficiency and at the same frequency, or the emission window. Usually in HHG systems, the emission window is determined by ionization gating, where phase-matching is lost due to the free-electron plasma generated in field ionization.^5,35,57^ The plasma phase shifts on the fundamental pulse gate the harmonic emission when they approach ∼*π*/*q*, where *q* is the harmonic order. However, in CE-HHG, because the power enhancement relies on the constructive interference of the pulse in the cavity with pulses coming from the laser, time-dependent phase shifts are restricted to be less than 2π/F,^58^ roughly one order of magnitude smaller than the scale relevant for ionization gating. Under these conditions, the emission window is determined by the high harmonic dipole, *d_q_*(*t*), and not ionization gating, such that harmonic linewidths significantly narrower than achieved in single-pass HHG systems under their typical operating conditions are expected. Indeed, Mills *et al.*^27^ have reported single harmonic linewidths of 32 meV from a cavity-enhanced HHG source with similar parameters to ours, even starting with shorter fundamental laser pulses. In our setup, more indication that the resolution of the photoelectron spectra reported here is limited by the hemispherical analyzer comes from experiments where we have intentionally lengthened the intracavity IR pulses by detuning the comb/cavity coupling^45^ and observed no changes in the EDC. In the absence of ionization gating, increasing the pulse duration increases the emission window. Further investigation of the attainable energy resolution and its potential manipulation will be the subject of future work as we upgrade our electron energy analyzer. Please see the supplementary material for additional discussion and calculations regarding the HHG physics.

**FIG. 6.**
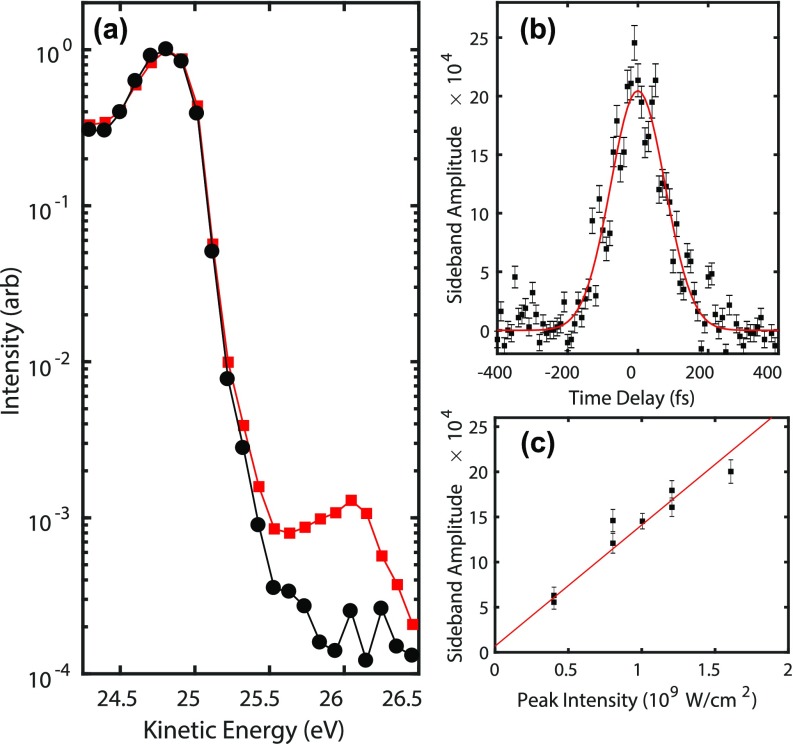
**(**a) The photoelectron spectrum of the Au (111) Fermi edge taken without (black) and with a 1.035 *μ*m pump pulse (red) at a peak intensity of 1.3 × 10^9^ W/cm^2^. A LAPE sideband of the surface state peak at 24.8 eV is observed at 26 eV. (b) The magnitude of the sideband at a kinetic energy of 26 eV as a function of pump probe time delay. The cross-correlation has a FWHM of 181 fs. (c) The amplitude of the sideband at 26 eV as a function of pump peak intensity. A fit to the data gives a slope of 1.34 × 10^−12^ cm^2^/W.

Even with the current analyzer-limited resolution, we demonstrate here that the absence of space-charge allows for time-resolved photoemission experiments that are both qualitatively and quantitatively different than what is done with space-charge limited systems. Figure 6 shows two photoelectron spectra near the Fermi edge of the Au (111) on a logarithmic scale, one with and another without a parallel polarized 1.035 *μ*m wavelength laser excitation overlapped in space and time. The spectra were taken with 3 nA of sample current, or approximately 215 electrons/pulse. Consider first the black curve taken with the pump laser off. For a 100 kHz system with our spot size (or even somewhat larger), this sample current would result in broadening and shifting of the Fermi edge on the eV scale instead of the ≲10 meV effects here estimated using Eq. (1). Furthermore, on a logarithmic scale, space charge effects can cause long high energy tails in the photoelectron spectrum^10,21,25^ that make it difficult to observe small signals from weakly excited samples. Here, with excellent harmonic isolation from our pulse-preserving monochromator and the absence of space charge effects, a precipitous drop of four orders of magnitude is observed in the EDC at the Fermi edge.

Next, consider the red curve taken with the pump laser on. Before discussing the laser-induced features of the spectrum, consider first what is *not* observed. The photoelectron spectrum is not shifted, broadened, or distorted due to space charge produced by the laser excitation as is commonly observed in pump probe experiments.^21,23–25^ This is because the high data rate enables the experiment to be performed under the low pump intensity of 1.3 × 10^9^ W/cm^2^. We measured the sample current from the pump excitation alone and found it to depend strongly on the region of sample probed, as in Ref. 21, but always at least one order of magnitude less than the XUV probe, or less than 22 electrons/pulse produced by the pump.

The reflectivity of the gold sample is >97% and the laser-induced features in the EDC are dominated by the now well-known laser-assisted photoelectric effect (LAPE).^21^ Briefly, LAPE is a dressing of the ionized electrons by the IR laser field producing sidebands at intervals of the photon energy.^21,59–61^ In Fig. 6(a), a sideband of the surface state peak at 24.8 eV is observed 1.2 eV higher at 26 eV. Figure 6(b) shows a measurement of the sideband amplitude at 26.0 eV kinetic energy as a function of the time delay between the IR pump and XUV probe. A cumulative integration time of 7.5 s for each time delay with 10 fs size steps results in a total scan time of 10 min. Within statistical error, identical widths and time-zero positions are observed for cross correlations taken at both higher and lower kinetic energies, further confirming the LAPE mechanism, as hot electrons closer to the Fermi energy would show observable lifetimes.^62,63^

LAPE can be used to determine the time resolution of the instrument. A Gaussian fit to the cross-correlation in Fig. 6(b) gives a FWHM of 181 fs. The pump laser pulse duration at the sample position was not independently measured for this experiment, but at the output of the laser the pulse duration was measured to be 165 ± 10 fs and optimal compression gives 155 fs.^44^ Taking the lowest possible value of the laser pulse duration then gives a conservative upper limit for the XUV pulse duration at the sample of $\sqrt{{\left(181\;\text{fs}\right)}^{2}-{\left(155\;\text{fs}\right)}^{2}}=93\;\text{fs}$.

Figure 6(c) shows amplitudes for the sideband at 26 eV kinetic energy for different pump pulse intensities obtained from fits to time-resolved scans as shown in Fig. 6(b), but with a 20 fs time delay step and 5 s integration time. The sideband amplitude is observed to be linear in the laser intensity with a slope of 1.34 ± 0.13 × 10^−12^ cm^2^/W, and in excellent agreement with theory (1.3 × 10^−12^ cm^2^/W) for our laser and experimental geometry, as calculated in Appendix B. Even with the modest time of 3.3 min used to acquire each data point in Fig. 6(c), sideband amplitudes as small as 6 × 10^−4^ with an uncertainty of 1 × 10^−4^ are easily observed. We also note that with a multichannel electron analyzer, the delay-dependence for the full energy window of Fig. 6(a) (or larger) could be obtained in parallel with no increase in data acquisition time.

## IV. CONCLUSIONS

Pump-probe experiments in condensed matter physics can broadly be divided into non-perturbative “high-fluence” and perturbative “low-fluence” experiments.^22^ High-fluence experiments study photo-induced phase transitions or non-thermally accessible meta-stable states. Low-fluence experiments, where the system is excited as “gently as possible,”^22^ study phenomena such as exciton dynamics or electron-photon coupling. While ARPES using tunable synchrotron radiation is often the method of choice for studying a material's electrons in their ground states, excited-state XUV ARPES studies have been limited to the high-fluence regime, where optical excitation produces substantial changes to the EDC visible on a linear scale. Currently, to study dynamics in the low-fluence regime, workers in the field turn away from the clarity and fidelity of XUV ARPES and instead pursue other methods that have the required sensitivity but are limited in scope and harder to interpret. For example, low-fluence experiments are typical for the more sensitive techniques of optical spectroscopy,^64^ two-photon photoemission,^65^ and laser-based ARPES using 6 eV probe light^66^ but have been extremely difficult using space-charge limited HHG systems.

In this work, we have shown how with high repetition rate, small laser-induced modifications of the photoelectron spectrum can be recorded without space-charge artifacts in reasonable acquisition times. We have observed LAPE with sideband amplitudes in the 10^−3^ to 10^−4^ range, orders of magnitude smaller than typical.^19,21,67^ To our knowledge, these are the smallest LAPE signals observed from a surface, but more importantly they mimic the response of weakly excited samples in the low-fluence regime where only a small fraction (i.e., 10^−4^ of the surface state) of the sample's electrons are excited, demonstrating the feasibility of low-fluence excited state XUV ARPES experiments.

It is important to note that with the appropriate photoelectron analyzer, the 88 MHz repetition rate of the XUV pulses also does not preclude high-fluence experiments. The photoelectrons are emitted from the sample in discreet bunches which can be either detected individually using time-of-flight (TOF) methods or gated using time-dependent voltages in the analyzer.^68^ Thus, if it is desirable to excite the sample at a lower repetition rate for high-fluence experiments or long-lived sample excited states, one can record photoelectrons from both short (fs) and long (ns) pump-probe delays simultaneously via time-resolved detection of the electrons. This has been implemented in synchrotron experiments.^69,70^ In this way, the repetition rate of the experiment is *only* limited by the recovery time of the sample, and one can tune the repetition rate to optimize the data rate for a given experiment. We also note that for excited state studies needing to record only a small energy window, the 88 MHz repetition rate is also ideally suited to state-of-the art TOF photoelectron analyzers, which can achieve orders-of-magnitude improvements over hemispherical analyzers.^1,68^ The trade-offs between sample current, repetition rate, energy window, and resolution are discussed in greater detail in the supplementary material.

Whereas space-charge sets a fundamental limit on most HHG-based photoemission instruments, the current time and energy resolution of the system do not represent any inherent limitations of the frequency-comb based methods used here and are instead limited simply by the laser pulse duration and energy analyzer performance. Both of these are straightforward to improve. For example, in our setup, sub-100 fs resolution could be obtained by leaving the XUV probe arm unchanged and implementing nonlinear pulse compression in the pump arm,^71,72^ which has no demands on the temporal coherence of the pulse train. CE-HHG can also be performed with shorter driving pulses,^73,74^ if desired. Single-grating pulse preserving monochromators have been shown to be compatible with temporal resolutions as small as 8 fs.^75^

Returning to Fig. 1, we use the conservative upper limit of 65 meV to compare the performance of our system against previous time-resolved photoemission work.^12,14,27–32^ As can be seen, the present work enables a dramatic improvement over space-charge limited systems operating at lower repetition rate. Notable also is the work of Jones and co-workers^27^ who have demonstrated to our knowledge the best resolution in HHG-based ARPES using a fixed photon-energy CE-HHG platform based on grating output coupling.^76^ However, the grating output coupling method makes dynamic harmonic selection difficult and also introduces larger pulse front tilts than the pulse-preserving monochromator, resulting in larger focused spot sizes and XUV pulse durations.

In this work, we have developed a light source that, at the sample, reproduces the parameters of tunable synchrotron radiation critical for the success of data-intensive surface photoemission experiments, but with pulse durations ∼1000 times shorter, effectively eliminating the data-rate limitations imposed on previous efforts by space charge. Upgrading the electron analyzer to a multi-channel version will allow frames of time-resolved ARPES measurements to be accumulated at rates comparable to or larger than that attained synchrotron beamlines. enabling experiments from perturbatively excited samples and a much fuller exploration of the multidimensional data set.

## SUPPLEMENTARY MATERIAL

See supplementary material for the data used to determine the experimental energy resolution, an estimation of harmonic linewidth in cavity-enhanced HHG, and a discussion of the suitability of this XUV source for use with modern TOF energy analyzers.

## APPENDIX A: COMPARISON OF SOURCES IN Fig. 1

Both the development of HHG sources and their application to surface photoemission have been very active fields of research.^12,14,27–32,35,77^ To our knowledge, this work represents the first tunable HHG source applied to photoemission with nA sample currents capable of achieving sub-100 meV resolution in a spot size less than 1 mm. In Fig. 1, we have attempted to compare this work to previous published efforts applying HHG to sub-ps time-resolved surface photoemission. A time resolution of 1 ps corresponds to a 2 meV Fourier limit to the energy resolution. The following criteria were made in choosing which results to include the following: (1) Only systems with photon energy greater than 10 eV were included. (2) Only systems capable of achieving more than 1 pA of sample current were included. (3) Only systems driven by lasers with sub-ps pulse duration, such that they could, in principle, perform sub-ps time-resolved experiments, were included. (4) Only systems with published reports of being applied to photoemission have been included.

For the energy resolution of photoemission experiments, several authors have shown that space charge broadening adds in quadrature with the photon energy bandwidth^10,12^

$$\Delta E=\sqrt{\Delta E_{b}^{2}+\Delta E_{h\nu }^{2}},$$ (A1)

with $\Delta E_{b}$ being the broadening due to space-charge and $\Delta E_{h\nu }$ being the photon energy bandwidth. The black dashed lines in Fig. 1 represent the asymptotes of Eq. (A1) with $\Delta E_{h\nu }\ll \Delta E_{b}$. On the comparison plot, systems were categorized as not being space-charge limited (red edges) if the full flux can be applied to the sample in a 1 mm spot with Δ*E*_b_ according to Eq. (1) at least one order of magnitude less than $\Delta E_{h\nu }$. For non-space-charge limited experiments, the y-ordinate is determined either from published operating sample currents or published photon fluxes at the sample by assuming a photoelectric yield of 0.1 electrons/photon. The x-ordinate is the published photon energy bandwidth.

For space-charge limited systems (black edges), the (x,y) positions represent the case where the sample current is such that $\Delta E_{b}=\Delta E_{h\nu }$, and the energy resolution is broadened by a factor of $\sqrt{2}$. Since achieving resolution equal to the photon energy bandwidth requires substantially reduced sample current, this represents a practical compromise. The y-ordinate is calculated $I_{\text{sample}}=ef_{\text{rep}}D\Delta E_{h\nu }/m_{b}$, with D = 1 mm. The x-ordinate is then $\sqrt{2}$ times the published photon energy bandwidth. For ARPES, experiments are usually run with spot sizes smaller than 1 mm,^78^ such that even lower sample currents than plotted on Fig. 1 are required to maintain energy resolution. Furthermore, we comment that for real-space imaging with photoemission electron microscopy, the space-charge constraints are even more severe.^79,80^

## APPENDIX B: THEORY FOR LAPE AMPLITUDE

The laser-assisted photoelectric effect can be considered the result of dressing of the free-electron wavefunction.^21,59,61^ In the limit of electron kinetic energies much larger than the dressing laser photon energy and ponderomotive energy much less than the photon energy, the amplitude of the *n*th sideband (*A_n_*) becomes (in atomic units)

$$A_{n}=J_{n}^{2}\left(\frac{p\cdot E_{0}}{\omega^{2}}\right),$$ (B1)

where **p** is the vector momentum of the electron, $E_{0}$ is the laser electric field vector amplitude at the surface, and *ω* is the laser frequency. The geometry of the experiment influences observed sideband amplitudes in two ways. First, energy can only be transferred between the laser field and the electron at the surface, such that for metallic surfaces only the component of the electric field normal to the surface contributes to LAPE.^21^ Second, due to the dot product in Eq. (B1), only the component of the electric field along the detection direction contributes. For our geometry, with the sample oriented 45° to incident p-polarized beam and electrons detected along the surface normal, these factors are one and the same, and in the limit that the argument of the Bessel function in (B1) is much less than one we have (in SI units)

$$A_{1}\approx \frac{4\pi \alpha IE_{\text{kin}}}{m_{e}\hbar \omega^{4}}\;{\text{cos}}^{2}45{}^{\circ},$$ (B2)

where *α* is the fine structure constant, *I* is the laser intensity ignoring the effects of the surface on the laser electric field, *E*_kin_ is the kinetic energy of the electron, *m_e_* is the mass of the electron, and ℏ is Planck's constant. In the experiment, the laser intensity at which free electrons are generated varies in both space and time due to the finite extent of the XUV and laser beams. The observed sideband amplitude will thus be the space-time average

$$\begin{array}{c} \left\langle A_{1}\right\rangle =\frac{2\pi \alpha E_{\text{kin}}}{m_{e}\hbar \omega^{4}}I_{\text{peak}}\int_{-\infty }^{\infty }dx\int_{-\infty }^{\infty }dy\int_{-\infty }^{\infty }dtG_{\text{laser}}(x,y,t)G_{\text{XUV}}(x,y,t), \end{array}$$ (B3)

where *I*_peak_ is the peak intensity of the laser, $G_{\text{laser}}(x,y,t)$ is a 3D Gaussian envelope function for the incident laser beam with unit amplitude, and $G_{\text{XUV}}(x,y,t)$ is a normalized 3D Gaussian envelope function for the XUV beam. Evaluating the space-time overlap integral with 1/*e*^2^ radii $w_{x,\text{laser}}=150\;\mu m,w_{y,\text{laser}}=150\;\mu m,w_{x,\text{XUV}}=49\;\mu m,w_{y,\text{XUV}}=85\;\mu m$, and FWHM pulse durations *T*_laser_ = 165 fs and *T*_XUV_ = 93 fs, the integral is 0.72 and the theoretical estimate for the observed sideband amplitude is $\left\langle A_{1}\right\rangle =1.3\times 10^{-12}\times I_{\text{peak}}[W/{\text{cm}}^{2}]$.
